# AN INNOVATIVE DATA-DRIVEN FALL PREVENTION COMMUNICATION TOOL FOR ADMINISTRATORS, NURSE MANAGERS, AND STAFF NURSES

**DOI:** 10.1093/geroni/igz038.1208

**Published:** 2019-11-08

**Authors:** Ragnhildur Bjarnadottir, Robert Lucero

**Affiliations:** University of Florida, Gainesville, Florida, United States

## Abstract

Falls are the leading cause of injury among older adults, resulting in 3 million emergency department visits and 800,000 hospitalizations each year in the United States alone. In the hospital setting, falls are among the most common adverse events, causing longer stays and higher costs of care. Substantial efforts have been made to reduce falls in the past decade but with limited sustained effect. This may in part be due to limitations of existing tools for fall-risk assessment and evidence-based fall prevention. Therefore, there is a need to strengthen the evidence on risk factors for hospital-acquired falls and address barriers to translating the best available evidence into practice. To this end, we undertook the development of an innovative dissemination tool to implement fall-related evidence generated through state-of-the-art data science and information visualization approaches. Through a multidisciplinary academic-clinical partnership, we have developed an infographic to disseminate empirical evidence to nurses and administrators in the hospital setting. The infographic was developed based on principles of user-centered design and persuasive communication, and focuses on providing clear and accessible information about factors contributing to a patients’ risk of falling in the hospital. This innovative dissemination approach is intended to foster dialogue between administrators, mid-level nursing management and staff nurses as well as evidence-based practice at the bedside. Future use and evaluation of this fall prevention tool will focus on adapting the infographic as an interactive digital tool for education and engagement of patients and families in the hospital setting.

